# From Nature to Technology: Exploring Bioinspired Polymer Actuators via Electrospinning

**DOI:** 10.3390/polym15194029

**Published:** 2023-10-09

**Authors:** Muhammad Yasar Razzaq, Maria Balk, Magdalena Mazurek-Budzyńska, Anke Schadewald

**Affiliations:** 1 Institut für Kunststofftechnologie und Recycling e. V., Gewerbepark 3, D-6369 Südliches Anhalt, Germany; 2 Institute of Active Polymers, Helmholtz-Zentrum Hereon, Kantstraße 55, D-14513 Teltow, Germany; 3 Department of Chemistry, Warsaw University of Technology, ul. Noakowskiego 3, 00-664 Warsaw, Poland

**Keywords:** electrospinning, bioinspired actuators, stimuli-sensitive hydrogels, shape-memory polymers (SMPs), electroactive polymers

## Abstract

Nature has always been a source of inspiration for the development of novel materials and devices. In particular, polymer actuators that mimic the movements and functions of natural organisms have been of great interest due to their potential applications in various fields, such as biomedical engineering, soft robotics, and energy harvesting. During recent years, the development and actuation performance of electrospun fibrous meshes with the advantages of high permeability, surface area, and easy functional modification, has received extensive attention from researchers. This review covers the recent progress in the state-of-the-art electrospun actuators based on commonly used polymers such as stimuli-sensitive hydrogels, shape-memory polymers (SMPs), and electroactive polymers. The design strategies inspired by nature such as hierarchical systems, layered structures, and responsive interfaces to enhance the performance and functionality of these actuators, including the role of biomimicry to create devices that mimic the behavior of natural organisms, are discussed. Finally, the challenges and future directions in the field, with a focus on the development of more efficient and versatile electrospun polymer actuators which can be used in a wide range of applications, are addressed. The insights gained from this review can contribute to the development of advanced and multifunctional actuators with improved performance and expanded application possibilities.

## 1. Introduction

Nature has long served as a boundless source of inspiration for scientists and engineers seeking to develop innovative technologies. The realm of bioinspiration, specifically, aims to emulate and harness the extraordinary capabilities observed in biological systems [1,2]. One area of particular interest in biomimetics is the development of polymer actuators inspired by nature [3]. Polymer actuators can convert electrical, thermal, or chemical energy into mechanical motion. They are composed of polymeric materials that can undergo significant changes in shape, size, or stiffness in response to a stimulus. This unique property makes them ideal for use in a wide range of applications, including (soft) robotics, biomedical devices, energy harvesting systems, and artificial muscles [4,5,6]. A well-known example of a bioinspired actuator is the artificial muscle, which aims to replicate the contractile behavior of natural muscles. Natural muscles are composed of a network of protein fibers that can slide past one another to generate force and motion [7]. Other examples of natural actuations that have inspired materials scientists include the movement of plant tendrils [8] and the morphing of butterfly wings [9]. Tendrils are able to wrap around support structures by combining changes in turgor pressure with differential growth, while butterfly wings can change their color and shape through the controlled movement of scales and ridges [10,11]. 

Researchers have developed various bioinspired polymer actuators (BiPAs) with unique properties and based on various smart polymers such as stimuli-sensitive hydrogels, shape-memory polymers (SMPs), or electroactive polymers (EAPs) [12,13,14,15]. One of the most notable features of these materials is their ability to undergo large, rapid changes in shape and size in response to stimuli such as temperature, humidity, light, or electric fields [5,6,16,17,18,19]. For instance, stimuli-sensitive hydrogels are hydrophilic polymer networks and exhibit large volume changes, leading to shape transformations or the generation of mechanical work by exposure to different stimuli [6,20,21]. SMPs are another fascinating class of smart materials, which possess the ability to undergo substantial deformation when subjected to an external stimulus such as heat, light, or pH change, and subsequently, recover their original shape upon exposure to a triggering stimulus [17,22,23,24,25]. EAPs, in contrast to SMPs, have the ability to deform under the influence of an electric field and are attractive for a wide range of applications including robotics, biomedical devices, haptic interfaces, and microelectromechanical systems (MEMS) [13,26,27]. Overall, the synthesis of these intelligent polymer materials, integration of multiresponsive functionalities, and progress in fabrication techniques have significantly expanded their capabilities and performance. 

In addition to their responsive properties, stimuli-sensitive polymers can also exhibit remarkable strength, flexibility, and durability [11,18,28,29,30]. This makes them highly desirable for applications such as soft robotics and wearable devices, where flexibility and durability are key requirements [31]. In recent years, significant advancements have been made in the synthesis, characterization, and application of these smart polymers, propelling them into exciting new territories. However, despite the many promising features of BiPAs, there are also significant challenges associated with their development and use. For example, the design and fabrication of these materials require careful consideration of factors such as scalability, biocompatibility, and stability over time. Additionally, there is a need for improved characterization techniques and theoretical models that can accurately predict the behaviour of these complex materials [18,31,32,33]. 

**Figure 1:**
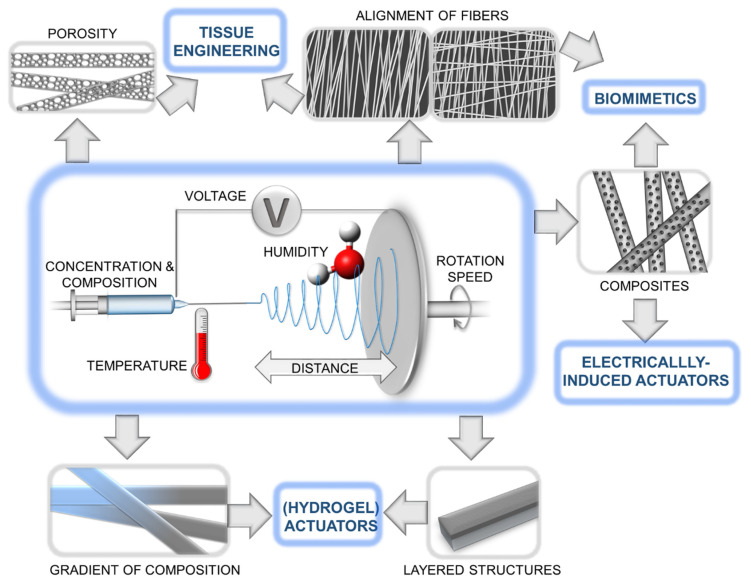
Schematic illustration of the electrospinning process along with various architectures of electrospun fibers and potential applications.

Electrospinning is a widely used technique for fabricating polymeric fibrous meshes, as it allows for precise control over the fiber diameter and alignment, as well as the incorporation of various functional components (Figure 1) [34,35]. The use of nature as a source of inspiration for the design of electrospun polymer actuators has led to the development of devices that can replicate the movements and functions of natural organisms. For example, some electrospun polymer actuators have been designed to mimic the movements of muscle fibers [36], while others have been inspired by the motion of insects, plants, or other organisms. By controlling the parameters of electrospinning, such as the solution viscosity, flow rate, and applied voltage, it is possible to tune the morphology and mechanical properties of the resulting fibers [37]. One of the advantages of electrospun polymer actuators is their ability to exhibit multiple functions, such as responding to various stimuli and performing multiple tasks such as locomotion, sensing, color changing, etc. [38]. Moreover, electrospinning can be combined with other techniques such as 3D printing and microfabrication to create complex and functional structures. 

As the field of bioinspired electrospun actuators continues to expand, several challenges and opportunities lie ahead. Researchers have published a number of review articles on polymer actuators based on stimuli-sensitive polymers or electrospinning of functional polymers in general along with characterization and applications of electrospun fibers [30,32,34,35]. However, these reports have not extensively addressed the actuation aspect of electrospun fibers. This paper will review the current advancements, fabrication techniques, and actuation mechanisms of three major categories of electrospun polymer actuators based on hydrogels, SMPs, and EAPs, while also discussing the limitations and potential solutions to overcoming them. By presenting an overview of the latest research, this paper aims to inspire and guide researchers in their pursuit of bioinspired polymer actuators, fostering a deeper understanding of the synergistic relationship between nature and engineering and unlocking new frontiers in the development of next-generation actuation technologies.

This review also highlights the potential applications of electrospun polymer actuators in different fields including tissue engineering, sensor technology, smart textiles, artificial muscles, and soft robotics. Finally, the challenges and future directions in the field of electrospun BiPAs inspired by nature will be discussed. These challenges include improving the efficiency and controllability of these devices, as well as expanding their potential applications in different fields. Overall, this review provides a comprehensive overview of the current state of the art in this exciting field and highlights the potential for the development of new and innovative fiber-based polymer actuators inspired by nature.

## 2. Biomimetic Inspiration for Synthetic Actuators

**Figure 2:**
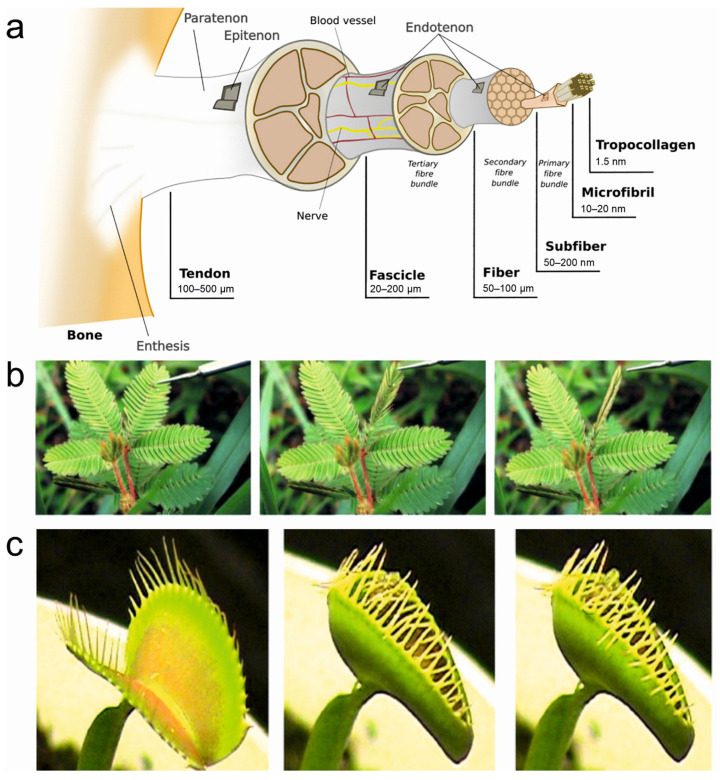
Representative actuating structures in living systems. (**a**) Hierarchical structure of the tendon/ligament. The tropocollagen crosslinked molecules are arranged in microfibrils, subfibrils, fibrils, fibers, and fascicles. Reprinted with permission from [45]. (**b**) Touch-induced folding/unfolding of mimosa leaflets. Reprinted with permission from [43]. (**c**) The snap–trap of the carnivorous Venus flytrap (*D. muscipula*) closes after the mechanical triggering of sensitive hairs, leading to a swift concave–convex curvature change in the two trap lobes. Reprinted with permission from [46].

Biological systems, honed by millions of years of evolution, offer an extraordinary repertoire of intricate and adaptive movements that have inspired the development of cutting-edge synthetic actuators. As researchers have delved deeper into the mechanisms governing stimuli-sensitive locomotion and movements in living organisms, they have uncovered fascinating adaptations that not only showcase remarkable performance but also unparalleled energy efficiency [14,30]. For instance, animals rely on muscle contractions for various movements, such as walking, flying, and swimming [39]. Muscles are composed of bundles of specialized fibers called myofibrils, which are made up of proteins called actin and myosin. When stimulated by electrical signals from the nervous system, actin and myosin interact, causing the myofibrils to contract and generate force. This contraction allows organisms to produce a wide range of reversible movements, such as walking, grasping, or flexing muscles [40,41]. Tendons and ligaments are fibrous connective tissues that attach muscles to bones (tendons) and bones to other bones (ligaments) (Figure 2a). They are primarily composed of collagen fibers, which provide strength and flexibility. Tendons transmit forces from muscle contractions to bones, allowing movement at joints, while ligaments provide stability and limit joint movements to prevent excessive strain [42]. Marine invertebrates possess specialized fibrous structures that enable reversible movements. For example, certain types of tentacles in jellyfish or anemones contain fibrous proteins that can rapidly extend or contract, allowing these organisms to capture prey or withdraw from potential threats [43]. Tropic or nastic responses in plants enable directional growth or movements to adopt new environments. For instance, tropic responses include phototropism, which is the movement of plants in response to light. Photoreceptor cells, such as phytochromes and phototropins, sense light intensity [44] and direction, thus triggering differential growth in various parts of plants [45].

Climbing plants, like vines, employ thigmotropism to sense and respond to physical contact [47]. They possess specialized fibrous structures, such as tendrils, which wrap around support structures when they come into contact with them [48]. By doing so, these plants can grow and climb upward, utilizing external structures for stability and support. The nastic movements in plants are reversible, non-directional responses to external stimuli that result in changes in the orientation of plant organs, e.g., rapid folding or drooping of leaves in the sensitive plant *(Mimosa pudica*) when touched. These movements are facilitated by specialized filaments or fibrous tissues within the plant structures, which undergo reversible changes in turgor pressure or cell elongation [46]. Another example is seismonastic movements that occur in response to vibrations or mechanical disturbances, e.g., the Venus flytrap, which closes its leaves rapidly when triggered by the movement of an insect [49]. There are tiny hairs at the edges of the leaves which act as mechanosensors and trap the insect by generating an electrical signal [50]. Some of the representative actuations in living systems are shown in Figure 2. The adaptability and responsiveness of living systems to different stimuli, which not only significantly enhance their survival abilities but also provide inspiration for the design of bioinspired actuators capable of responding to a range of stimuli, open doors to innovative applications in robotics, materials science, and biomedical engineering [1,14,32].

## 3. Hydrogel Actuators Created by Electrospinning

Many natural and artificial materials can show swelling and shrinkage behavior by responding to changes in environmental humidity. As examples, the skin of the human hands and feet forms wrinkles upon continued submersion in water [51] and pine cones can exhibit a folded shape on rainy days and an open shape when it is dry [52]. Especially in the case of pine cones, this motion relies on a bilayered structure of the individual scales that can change their conformation when the humidity is increased/decreased [53].

This phenomenon inspired the design of bilayer hydrogel actuators, in which one of the layers (called the active layer) can change its volume by means of swelling/deswelling effects [21]. The second layer (called the passive layer) is inert to the dimensional change. A macroscopic shape shift can be obtained as a result of generated stresses at the interface of the two layers. The resulting movements can be controlled by the variation in material types, thickness, mechanical characteristics in the bilayer system, sample size, and geometry. Electrospun hydrogel actuators are often based on thermoresponsive polymers (e.g., poly(*N*-isopropylacrylamide)—PNIPAM), which can uptake or release water as a function of temperature based on a phase separation upon heating (lower critical solution temperature—LCST) [54,55]. Although organic solvent-based synthetic actuators are described in the literature, water-based systems are of importance as water is highly relevant for living systems and biomedical applications [18]. Hence, the research on electrospun actuator materials has also focused on hydrogel-based systems. In Table 1, an overview of the different types of stimuli to initiate directed movements in electrospun hydrogel actuators, their material composition, the characteristics of actuator morphology, electrospinning conditions, and possible applications are presented.

### 3.1. Hydrogel Actuators with Water and Temperature Sensitivity

Reversible movements of electrospun hydrogels were reported by a humidity-responsive bilayer actuator, which was created by combining an electrospun polyvinylpyrrolidone (PVP)/poly(acrylic acid) (PAA) active layer with a polyimide film as the passive layer [56]. This actuator showed bending movements when humidity was increased as result of swelling within the active layer and the created stress mismatch for the bilayer system. In this case, the properties of the active layer changed during actuation between a swollen hydrogel and a non-swollen polymeric layer, which presents the simplest concept for the design of reversible movements for electrospun hydrogel actuators. 

A temperature-triggered swelling/deswelling response with increasing/decreasing sample volume was realized by electrospun hydrogels based on the copolymer of thermoresponsive NIPAM with a photoreactive 4-acryloylbenzophenone (ABP)—conjugated comonomer. The created crosslinked copolymer mats showed a rapid swelling behavior in an aqueous environment with a water content >60 wt%. As result of the LCST of PNIPAM, aggregates based on hydrogen bonding as additional temporary crosslinks can be created in hot water (deswelling). The prepared fibrous hydrogels showed a fast thermal response and a rapid self-recovery (74% within 10 s) after loading–unloading tensile cycles in water. Besides changes in material volume, real reversible movements were reported by hydrogels with a super-fast actuation behavior of less than one second [57]. Here, bilayer nanofiber mats made by sequential electrospinning and subsequent photocrosslinking of the thermoresponsive polymer P(NIPAM-ABP) (active layer) and thermoplastic polyurethane (TPU—passive layer) were designed. A macroscopic shape shift was realized in warm water (40 °C) and the restoration of sample shape, in cold water (4 °C). By means of self-folding triggered by temperature changes, 3D structures could be obtained. 

**Figure 3:**
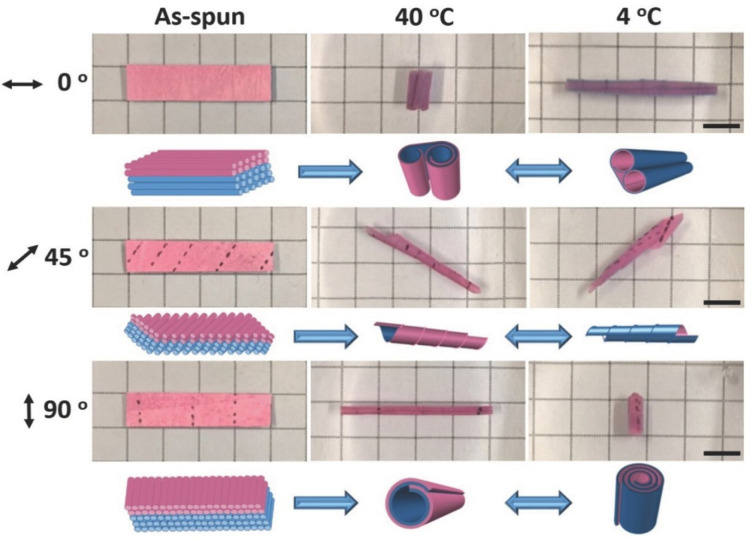
Actuation behavior of fibrous bilayer systems with fiber orientation-dependent shape shifts. Reprinted with permission from [59].

One concept to adjust the bending movement for electrospun soft actuators is based on the variation in the thickness of the different layers as demonstrated for PNIPAM and cellulose nanocrystal (CNC) bilayer actuators [58]. The two layers included different amounts of CNCs, whereby the water uptake upon heating and cooling was influenced (decreased swelling with increased CNC content). A 3D geometry was obtained when the bilayer system came into contact with water for the first time via anisotropic swelling of the two layers. Reversible shape shifts were observed by subsequent swelling/deswelling as a result of temperature changes between 20 °C and 40 °C. More interestingly, the direction of macroscopic shape shifts can be controlled. As an example, reversible shape shifts could be realized by means of a fibrous bilayer system with TPU (as the passive layer) and crosslinked PNIPAM fibers (as the active layer) [59]. Here, the angle of fiber orientation controlled the direction of reversible movements. The bilayers were developed by the sequential electrospinning of TPU with ABP as the photocrosslinker and of the thermoresponsive PNIPAM with ABP. The direction of actuation behavior was adjusted by cutting the bilayer systems at different angles (0° fibers oriented parallel to the long axis; 90° fibers oriented perpendicular to the long axis; and 45° fibers oriented 45° to the long axis). The asymmetric swelling (at 4 °C)/shrinking (at 40 °C) of hydrogel fibers as a result of temperature changes induced the creation of different shapes and the high porosity of the bilayer system enabled a fast actuation (Figure 3).

As an alternative to that, the combination of electrospinning of a thermoresponsive PNIPAM membrane and 3D printing of different well-designed PNIPAM/clay patterns on electrospun membranes enables for the created internal stresses generated by swelling/deswelling effects to be guided [60]. Hence, complex shape geometries with controlled shape shifts could be obtained. 

It has to be noted that most of the presented examples showed an actuation behavior based on swelling and deswelling effects, causing a stress mismatch in bilayer systems. Complex shape shifts can be obtained by cutting bilayer systems at different angles or by combining electrospinning and 3D printing, thus enabling macroscopic movements controlled by processing parameters (e.g., alignment of fibers). By contrast, SMPs can perform complex shape shifts by programming them into versatile temporary shapes (without requiring new synthesis). However, such electrospun hydrogel actuators, which provide shape-memory properties and are swollen during the whole shape-memory cycle, have not been reported so far. Shape-memory hydrogels often include hydrophobic crystallizable switching segments [61,62]. Only a few studies related to electrospun SMPs based on hydrophilic crystallizable switching segments have been reported [63,64]. Here, polymer was stretched or folded in a swollen state in water and in a subsequential drying step, crystallization of the switching domain (e.g., PEG) enabled the fixation of the temporary shape. The recovery to the original shape could be initiated, when a high amount of water was absorbed, resulting in the complete dissolution of the crystalline domain in water.

### 3.2. Light- and Electric Field-Responsive Hydrogel Actuators

**Figure 4:**
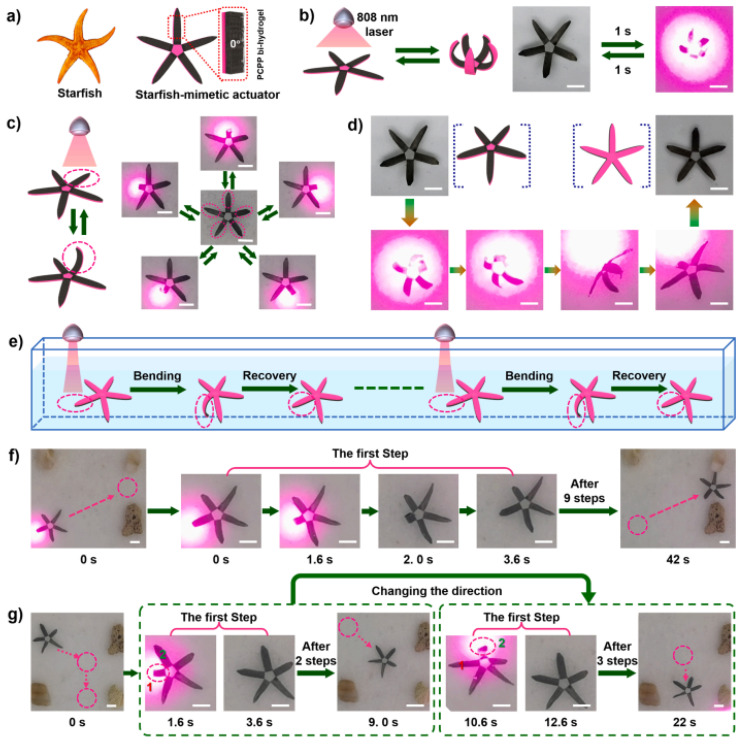
The starfish-mimetic actuating device. (**a**) The anisotropic structure of the starfish-mimetic actuating device. (**b**) The synchronized upward bending of the legs of the starfish. (**c**) The one-by-one bending of the starfish legs. (**d**) The turning-over process of the starfish. (**e**) Illustration of the continuous crawling movement of the starfish. (**f**) The continuous forward crawling movement and (**g**) the crawling movement in different directions of the starfish. Reprinted with permission from [65].

Besides water and heat as stimuli to induce an actuation function, light-responsive electrospun hydrogel actuators inspired by the hierarchical structure of a whale baleen have been reported [65]. The actuators were designed via in situ polymerization of pyrroles on nanofiber-oriented electrospun and temperature-responsive P(NIPAM-ABP) hydrogels. The coating with polypyrroles on nanofibers enhanced mechanical strength (from 1.21 to 5.12 MPa of tensile strength) and enabled ultrahigh-efficiency of photothermal conversion. As a result of the porous structure of hydrogel nanofibers (high specific surface area), the speed of the light-responsive actuation could be accelerated. When the ultrathin hydrogel layer (15 ± 3 µm) was bonded to a polyethylene glycol diacrylate-cellulose nanofiber (PEGDA-CNF) composite hydrogel membrane by means of interfacial UV polymerization, an anisotropic bihydrogel actuator was obtained. This actuator showed various programmable complex deformations with a powerful force (it could grab up to 100 times its weight), rapid speed (1285.71°/s of folding, 32.73°/s of bending, and 434.36°/s of bending recovery), and was utilized to imitate a continuous crawling movement of a starfish (Figure 4). As an additional stimulus, electric current was used to initiate reversible movements in hydrogel actuators based on fibers from acrylic acid (AA), PEG-diacrylate, acrylamide (AAM), and PVA that were in situ photopolymerized together with a polyaniline formation [66]. The obtained hybrid hydrogel mats showed high electric conductivity. A fast actuation behavior (2.5 mm·s^−1^) could be reached by applying low voltage (1 V) and low current (5 µA). By coupling a load cell to the electrochemical cell, generated forces could be monitored, which reached about 80 µN. 

### 3.3. Hydrogel Actuators with Multistimulus Response

In order to imitate the motion of soft materials such as living organisms, which act in an environment where multiple stimuli are simultaneously present, hydrogel actuators providing a multistimulus response were designed. When, e.g., a P(NIPAM-ABP) layer and a Fe_3_O_4_/polyacrylonitrile (PAN) layer are combined to obtain an anisotropic bilayer hydrogel actuator, multistimulus-responsiveness with programmable bifunctional synergistic movements can be realized [67]. Here, a bilayer structure was developed by the electrospinning of a PAN solution doped with Fe_3_O_4_ nanoparticles and a solution based on precursors for P(NIPAM-ABP). After crosslinking, a composite hydrogel, which was equipped with a thermoresponsive (P(NIPAM-ABP)) and a magnetoresponsive layer (Fe_3_O_4_/PAN), was obtained. The Fe_3_O_4_/PAN layer could perform long-range directional navigation highly controlled by a magnetic field on account of the doping with magnetic nanoparticles. In addition, the efficiency of the Fe_3_O_4_ nanoparticles endowed the P(NIPAM-ABP) layer with fast remotely controlled photothermal-responsive deformations (178°/s) when NIR light was applied. Here, the resulting swelling/deswelling effects triggered macroscopic movements. 

Depending on the functional groups in the hydrogel, the swelling behavior could also be dependent on pH. In this case, any pH-responsive group (e.g., acids, amines, or pyridines) in a hydrogel matrix can initiate pH-responsive swelling effects by deprotonation or protonation. As an example, thermo- and pH-responsiveness was realized in electrospun poly(NIPAM-co-AA) fibers, which were embedded within a passive TPU matrix [68]. The resulting composite had a gradient of the TPU along the thickness. At low pH value, the size of the demonstrator was controlled by the temperature (swelling at 6 °C, shrinkage at 40 °C). When the pH value was changed from pH = 3 to pH = 10, AA moieties were deprotonated and the generated charge repulsion increased the swelling. As a result, directed movements were obtained based on generated stresses in the composite and the angle, at which point fibers were embedded in the matrix that controlled the actuation direction. In addition, thermo- and pH-responsiveness was demonstrated by multilayer hydrogel actuators, which were fabricated by combining electrospinning and hydrogel lithography [69]. The combination of stimulus-responsive hydrogel fibers based on PAA and/or PNIPAM as the active layer(s) with a PCL-based passive layer by a micropatterned hydrogel coupling layer caused the reversible movement of the resulting actuators when the pH or temperature was changed. Here, shape shifts were regulated by modulating the mechanical properties of the actuator materials and dimensions of the hydrogel micropatterns. Utilizing the presented actuating system, artificial muscles providing holding, grabbing, transferring, and releasing behaviors as prototypes were fabricated. These examples show that easy incorporation of composite materials or changing the type of active layer enables the design of multiresponsive hydrogel actuators. Hence, prototypes of these multistimulus-sensitive hydrogel actuators could act in an environment where numerous stimuli are present in a predefined manner.

**Table 1:** Overview of electrospun hydrogel actuators where different stimuli triggered directed movements.

Stimulus	Materials and Solvents	Morphology	Electrospinning Conditions	Application	Ref.
Water	PVP/PAA active layer; polyimide film passive layer; Solvent: DMF	Fiber diameter: 0.91–1.14 µm; thickness active layer 18–47 µm and passive layer 55 µm	Voltage: 8–10.2 kV; feed rate: 0.8 mL/h; distance: 15 cm; rotational speeds: 180–2000 rpm	Robotics, energy harvesting, sensors	[56]
	PCL/PEG polyurethane linked with lysine methylester diisocyanate; Solvents: THF/DMF	Fiber diameter: 810 nm; thickness: 0.38 mm	Voltage: 13 kV feed rate: 0.3 mL/h; distance: 10 cm; rotational speed: 400 rpm	Water-responsive sensors, medical devices	[63]
	PCL/PEG/PDMS polyurethane linked with 1,6 hexamethylene diisocyanate; Solvent: HFIP	–	Voltage: 10 kV; feed rate: 0.5–1 mL/h	–	[64]
Heat	NIPAM-ABP; Solvent: DMF	Fiber diameter: 1.1–1.3 µm; thickness: 0.2 mm	Voltage: 15 kV; feed rate: 0.3 mL/h; distance: 15 cm; temperature: 25–30 °C; humidity: 15–30%	Artificial skin, smart separation membranes, tissue engineering	[20]
	P(NIPAM-ABP) active layer, polyurethane (Desmopan DP 2590) passive layer; Solvent: DMF	Fiber diameter: passive layer 238 nm and active layer 477 nm; thickness: passive layer 40 µm and active layer 15–100 µm	Voltage: 22 kV (passive layer) and 10.9 kV (active layer); feed rate: 0.6 mL/h; rotational speed: 30 rpm	Porous 3D bioscaffolds, electrodes, superfast actuators	[57]
	P(NIPAM-ABP) with CNC; Solvents: DMF/formamide	Fiber diameter: 0.14–0.17 µm	Voltage: 24 kV; feed rate: 0.3 mL/h; distance: 20 cm	Tissue engineering	[58]
	P(NIPAM-ABP) active layer, polyurethane (Desmopan DP 2590) passive layer; Solvent: DMF	Fiber diameter: passive layer 501 nm and active layer 1368 nm; thickness: passive layer 18 µm and active layer 53 µm; porosity: 56%	Voltage: 18 kV; feed rate: 1.3 mL/h; distance: 20 cm; rotational speed: 850 rpm	–	[59]
	P(NIPAM-ABP); Solvent: DMF and 3D printed PNIPAM/Laponite XLG patterns	Fiber diameter: 1.3 µm; thickness: 86 µm; porosity: 40%	Voltage: 25 kV	–	[60]
Light	P(NIPAM-ABP) active layer; Solvent: DMF; Coating of the active layer using APS and pyrrole; PEG-cellulose nanofiber hydrogel as passive layer	Fiber diameter: 560 nm; thickness: 15 µm	Voltage: 14.2 kV; feed rate: 0.6 mL/h; distance: 14 cm; rotational speed: 1000 rpm	Biomimetic devices (starfish’s crawling movement)	[65]
Electricity	AA, AAM, PEG, PVA, aniline; Solvent: water	Fiber diameter: 120–520 nm	Voltage: 15 kV	Wearables and soft robots	[66]
Heat and NIR light	P(NIPAM-ABP) active layer and PAN/Fe_3_O_4_ passive layer; Solvent: DMF	Fiber diameter: active layer 0.83 µm and passive layer 0.24 µm; thickness: active layer 150 µm and passive layer 50 µm	Active layer: voltage: 13.4–15.7 kV; feed rate: 0.8 mL/h; distance: 15 cm; rotational speed: 1000 rpm Passive layer: voltage: 16.2–19.4 kV; feed rate: 1 mL/h; distance: 20 cm; rotational speed: 20 rpm	Smart materials, bio-mimetic systems	[67]
Heat and pH	P(NIPAM-AA-ABP) active layer; Solvent: DMF polyurethane; (Desmopan DP 2590) passive layer; Solvent: THF	Fiber diameter: 905 nm; thickness: active layer 35.5–37.6 µm and passive layer 16.2–18.6 µm	Voltage: 14.3 kV; feed rate: 0.66 mL/h; distance: 8 cm; rotational speed: 1200 rpm	Sensors, artificial muscles, biomedical applications	[68]
	PAA/EGDE active layer; Solvent: ethanol; PNIPAM 2 active layer; Solvent: THF/DMF; PCL passive layer; Solvent: trifluoroethanol; Layers were soaked in a crosslinkable PEG solution	Fiber diameter: active layer 392 nm and passive layer 1.28 µm nm; thickness: active layer 26.8 µm and passive layer 112.3 µm	Voltage: 7 kV; feed rate: 1 mL/h; distance: 13 cm; temperature: 25 °C; humidity: 50%; rotational speed: 1200 rpm	4D scaffolds, artificial muscles, biomedical devices, soft robots	[69]

Abbreviations: PVP: polyvinylpyrrolidone; NIPAM: *N*-isopropylacrylamide; PCL: poly(*ε*-caprolactone); PAA: poly(acrylic acid); HFIP: 1,1,1,3,3,3-hexafluoropropan-2-ol; ABP: 4-acryloylbenzophenone; CNC: cellulose nanocrystals; APS: ammonium peroxodisulfate; AAM: acrylamide; PVA: poly(vinyl alcohol); AA: acrylic acid; PAN: polyacrylonitrile, EGDE: ethylene glycol diglycidyl ether; NIR: near-infrared.

## 4. Electrospun Shape-Memory Polymer (SMP) Actuators

A number of biological systems exhibit a shape change process when exposed to a wide variety of environmental changes, thus enabling sensing and responsive functionalities [12,70,71,72]. Synthetic materials can be programmed to intrinsically respond to environmental changes in a similar manner and have the potential to revolutionize materials science. One such example is shape-memory polymers, which can recover their original shape after being exposed to an external stimulus, such as heat, light, or a magnetic field [17,23,24,73]. These materials are unique as they can undergo a transition between a temporary shape and a permanent shape. The polymer is first programmed into a high-energy temporary shape by using a specific programming procedure. By exposure to an external stimulus, it reverts to its low-energy permanent shape. These polymers have attracted significant attention in recent years due to their potential use in a wide range of applications, including biomedical devices, aerospace, automotive, and textiles [17,22,23,33,61]. Recent studies have focused on the development of shape-memory electrospun fibrous meshes with actuation capabilities. These fibrous meshes have shown interesting advantages over bulk materials such as a faster recovery process, due to their high surface area (in the case of a thermally triggered process) and faster diffusion (in the case of a water-triggered process) [72,74]. They have also shown relatively high values of shape fixity and shape recovery ratios, with values ranging between 80% and somewhat higher than 90%. These benefits enable a versatile and customizable platform for developing advanced materials with enhanced mechanical, sensing, and actuation properties for various applications, including biomedical devices, smart textiles, and aerospace components [74]. However, most of these electrospun fibrous meshes are limited to a single, irreversible change in shape or pore size and the application of an external stress is required to program the sample [75]. Only few reports have dealt with reversible actuations or programmable pore size changes under stress-free conditions in covalently or physically crosslinked electrospun fibrous meshes. In Table 2, an overview of the different types of thermosensitive SMP actuators, their material composition, morphology, electrospinning conditions, actuation capability, and possible applications are presented. 

### 4.1. Covalently Crosslinked Electrospun SMP Actuators

**Figure 5:**
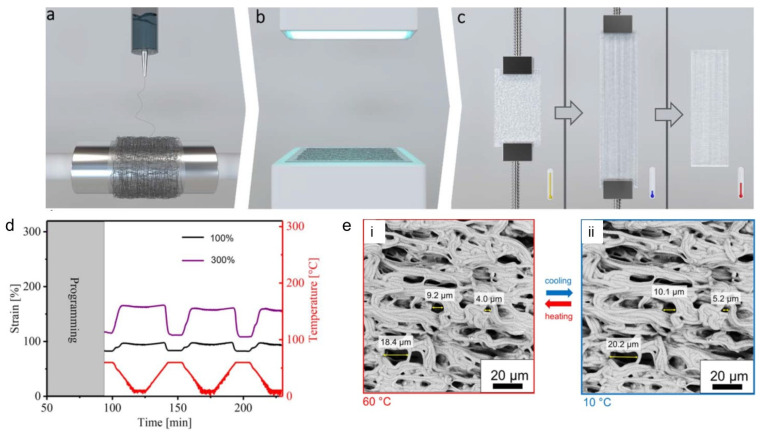
Schematic illustration of the fabrication and programming of electrospun fibers: (**a**) electrospinning; (**b**) crosslinking; and (**c**) programming. (**d**) Cyclic mechanical tensile tests (between 60 °C and 10 °C) of cPCL meshes with strains of 100% (black curve) and 300% (purple curve). The temperature curve is shown in red. (**e**) SEM image of cPCL heated to 60 °C (**i**) and after cooling at 10 °C (**ii**); exemplary pore diameters in the direction of deformation are shown. Reprinted with permission from [76].

Electrospun SMPs based on covalently crosslinked poly(ε-caprolactone) (cPCL) fibrous meshes enabled a bidirectional reversible actuation and a temperature-controlled reversible change in porosity [76]. A two-step preparation procedure was used to achieved the cPCL fibrous meshes. In the first step, the electrospinning of a blend of PCL with triallyl isocyanurate (TAI) and benzophenone (BP) was carried out, while in the second step, the electrospun fiber meshes underwent a photoinitiated crosslinking process. To observe the actuation behaviour, the meshes underwent deformation at an elevated temperature and cooling while keeping the deformation strain. A schematic demonstration of the electrospinning, crosslinking, and programming is provided in Figure 5a–c. After the programming step, the actuation of the meshes was triggered by heating and cooling between 10 °C and 60 °C as shown in Figure 5d. The actuation performance of the cPCL fibrous meshes was dependent on the programming strain. For a programming strain of 100%, a reversible microscopic reversible actuation strain of ε’_rev_ = 6 ± 1% was measured, while it was increased to 22 ± 1% for a programming strain of 300%, as shown in Figure 5d. The actuation performance was significantly higher in the meshes (ε’_rev_ = 15%) as compared to pure cPCL film with the same strain, and this was attributed to the higher orientation of the molecular chains through the electrospinning process.

Furthermore, SEM was carried out to observe a reversible change in the pore sizes at different temperatures under stress-free conditions. An apparent change in individually measured pore size of 11 ± 3% during actuation, from an average value of 10.5 ± 0.5 µm at 60 °C to 11.8 ± 0.6 µm at 10 °C, was obtained during an in situ measurement process as shown in the Figure 5e. 

**Figure 6:**
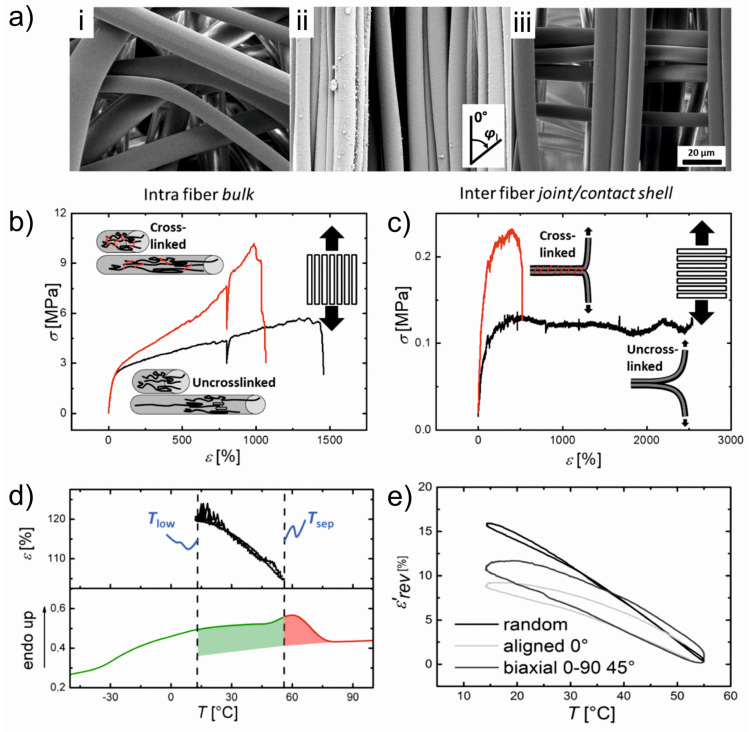
(**a**) SEM images of PEVA fiber meshes after an electrospinning process at RT with different fiber arrangements: (**i**) random; (**ii**) aligned; and (**iii**) stacked 0–90°. (**b**,**c**) Mechanical properties of aligned fiber bundles at RT of the crosslinked (red curves) and uncrosslinked (black curves) fibers as well as a schematic description of the molecular processes at different strains. The red color utilized in the schemes denotes covalent crosslinks. (**b**) Loading along the fiber direction and (**c**) perpendicular to the fiber direction. (**d**) Actuator capability after implementation of a thermomechanical programming procedure. Upper: Reversible elongation and contraction by cooling and heating between *T*_low_ and *T*_high_. Lower: DSC curve showing the broad thermal transition of the crosslinked electrospun fiber mesh scaffold. The curve is separated *T*_sep_ = 55 °C into green and red colors. Green: melting temperature range related to actuation units; Red: melting temperature range related to geometry-determining units. (**e**) Reversible actuation strain *ε՜*_rev_ depending on different fiber alignments after the samples were programmed. The original data points were smoothed by adjacent averaging. Random: black curve; Biaxial (0–90°) (45°): gray curve; Aligned 0°: light gray curve. Reprinted with permission from [77].

To see the effects of different spatial arrangements of stacked fiber bundles on the actuation behaviour, electrospun fiber meshes based on poly(ethylene-*co*-vinyl acetate) (PEVA) with different crosslinking densities were fabricated [77]. UV-based crosslinking was conducted to affect the interfiber bond strength by using TAI as a crosslinker and BP initiator. The SEM images of fiber mesh actuators with different fiber alignments (random, aligned, stacked 0–90°) are shown in Figure 6a. To prove an interfiber or intrafiber crosslinking, the tensile testing of the aligned PEVA fibers in the crosslinked or uncrosslinked state along the fiber direction (δ = 0° to observe the intrafiber bulk properties) and perpendicular to the fiber (δ = 90° to evaluate the interfiber bonds) was carried out. The crosslinking resulted in an increase in peak stress and decrease in elongation at break for both cases, indicating the presence of both interfiber and intrafiber crosslinking (Figure 6b,c). The actuation performance found in all fiber mesh geometries comprised an *ε*’_rev_ of 17 ± 2% for random fibers, 10 ± 1% for aligned fibers along the fiber (δ = 0°), and 12 ± 1% for 0–90° biaxial meshes in δ = 45°. The improved actuation capability in random meshes was attributed to an increased shape deformation in the initial heating to *T*_sep_ from the temporary shape *ε*_u_ = 148% to *ε*_T,sep_ around 105%, while *ε*’_rev_ varied below and above the programming strain *ε*_ssp_ = 150% for aligned fiber meshes (*ε*_u_ = 150%, *ε*_T,sep_ = 144%). The stronger initial shape deformation of the random mesh could be related to a microstructural as well as molecular aspect (Figure 6d,e).

### 4.2. Physically Crosslinked Electrospun SMP Actuators

To avoid the secondary crosslinking step by exposure to UV light, in situ crosslinking of electrospun fibers based on stereocomplexation was studied. Blending of a multiblock copolymer containing poly(L-lactide) and poly(*ε*-caprolactone) segments (PLLA-PCL) with oligo(D-lactide) (ODLA) in a one-step solution-casting process resulted in the formation of a stereocomplex [78]. This physical networking between the opposite enantiomers of PLA organized them into a periodic crystalline structure. The actuation behavior of the electrospun meshes and the bulk material was tested. The electrospinning process resulted in a higher molecular orientation, enabling an improved shape-memory actuation performance in the microfiber meshes compared to the bulk material. The actuation magnitude of *ε*_rev_ = 5.5 ± 0.5% in bulk PLLA-PCL/ODLA blends was increased to 7.8 ± 0.8% in fibrous meshes. Twisting the electrospun fibers into a yarn resulted in a further increase of actuation to 15 ± 1.8%. This was attributed to the improvement in the structural integrity of the fibers. The simultaneous orientation of polymer molecules and their crosslinking into a physical network opens a broad perspective for the development of actuating fiber meshes.

## 5. Electrospun Electroactive Actuators

Actuators based on electroactive polymers (EAPs) can operate when stimulated by an electric field. Due to the high surface-to-volume ratio of nanofibers and the porous structure of nanofibrous meshes, electrospun nanofibers have led to the fabrication of high-performance and versatile actuators [79,80,81]. One of the most promising applications of such devices is artificial muscles, which can mimic the function of natural skeletal muscles, and therefore, have potential applications in biomimetic robots and biomedical devices [5,7]. In recent years, various nanomaterials have been utilized to obtain artificial muscles and improve their mechanical, electrical, and electrochemical properties [82,83]. In Table 3, an overview of electrospun actuators based on electroactive polymers (EAPs) and liquid crystalline elastomers (LCEs) is presented. 

Actuation of such devices can happen according to two mechanisms based on electromechanical and electrochemical reactions. Electromechanical (electric) actuation is related to electric dipole rearrangements in electroactive polymers that cause dimensional changes, while electrochemical mechanisms utilize electrostatic forces, in which the ion exchange mechanism is responsible for volume changes in ionic electroactive EAPs [84,85]. In comparison to electromechanical, ionic EAPs offer a significantly lower voltage (1–2 mV); therefore, they can find applications in the medical field, especially in the preparation of artificial muscles. Furthermore, in the case of ionic EAPs, dimensional changes remain stable even after cutting off the electrical potential. To return to initial dimensionthe reverse potential is required. Furthermore, the wireless electrochemical actuation of ionic EAP-based artificial muscles has been investigated, which could eliminate the need for a direct physical connection to an electric power supply [16,86].

Much attention has been paid to the fabrication of conductive polymer-based actuators having nanofibrous structures [87,88]. However, direct fabrication of conducting polymer nanofibers using the electrospinning process is challenging due to the fact that typically, polymers with a low molar mass that are produced show inherent brittleness, as often the corresponding monomers have poor solubility in common solvents [89]. Therefore, several alternative approaches have been utilized to incorporate conducting polymers (CPs) into fibrous structures. The most common are the following: co-axial electrospinning to produce core–shell nanofibers and preparation of electrospinnable blends of polymers with CPs, which can be processed by electrospinning [90,91,92]. However, they result in the formation of a nanofibrous layer with low electrical conductivity and poor electrochemical properties for use as an actuator. A direct polymerization of CPs on the surface of substrate nanofibers is a simple and versatile technique for producing CP nanofibers of a core–shell structure [93]. Therein, a conducting polymer layer is on the surface of the fibers, which provides an effective nanofiber-conducting polymer-based actuator [94]. Furthermore, it was found that a multilayered cylindrical structure of nanofibrous mesh increases the active surface area of the actuator and thus facilitates the diffusion of ions [95].

Furthermore, core–shell nanofibers were prepared by gas-phase polymerization of PEDOT on the surface of PU electrospun fibers and were found to increase the distance actuated by CP actuators, whereas the addition of graphene nanoplatelets to PU-PEDOT nanofiber mats reduced actuator displacement because of a high modulus [96].

### Electroactive Actuators for Manufacturing Artificial Muscles

For the purpose of electrospun electroactive actuators, biopolymers such as cellulose [97], cellulose acetate (CA) [27], gelatin [98], chitosan [99], and silk [100] have been considered, particularly in biological and medical applications [95]. In most cases, biopolymer-based nanofibrous substrate is intended to increase the biocompatibility of an actuator. However, some biopolymers, such as CA, have naturally electrical actuation ability, which due to their synergistic effect with the active component, improve the final actuation performance of bioactuators [16,101]. Furthermore, due to their doping ability, CPs such as polypyrrole (PPy) [102,103], poly(3,4-ethylenodioxythiophene) (PEDOT) [94], and polyaniline (PANI) [98,104] can be used in conjunction with biopolymers to produce fibrous bioactuators for in vitro or in vivo applications, especially artificial muscles [32,105]. The chemical structure of CPs can expand and contract due to a flux of ions/solvent in and out of the polymer matrix when the conjugated backbone is electrochemically oxidized and reduced when low voltages are applied (1–2 V) in the presence of an electrolyte [106]. 

Beregoi et al. reported metalized PMMA fibers with good transparency, which were applied as a working electrode for PANI shell deposition by electrochemical polymerization. By controlling the potential applied, the oxidation state as well as the color of PANI was easily changed in the presence of an electrolyte. Moreover, biological investigations have revealed a good compatibility with eukaryotic cells for all PANI-coated samples [107]. Also, the preparation and actuation ability of PANI/Au microtubes was reported. It was found that such aligned PANI-coated microtubes with an inner diameter in the range of microns presented a muscle-like behavior, expanding and contracting by switching their potential between −0.2 and 1 V. The bending took place with a response time lower than 10 s [108]. Gu et al. reported a biomimetic myofibril that was made from parallel-oriented high-strength polyurethane (PU) and then chemically polymerized with aniline [109]. 

**Figure 7:**
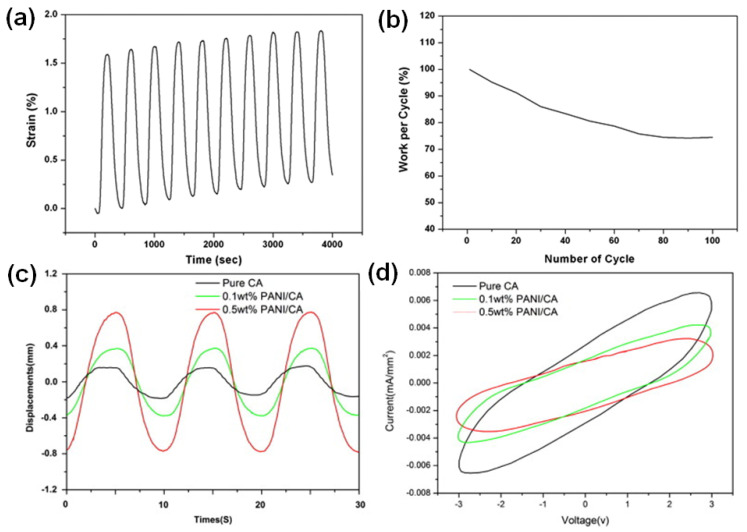
(**a**) Electrochemical actuation profile of a PU/PANI nanofibrous bundle upon cycling its potential between −0.2 to 0.8 V in an aqueous solution of 1 M MSA (scan rate = 5 mV/s, stress = 1.028 MPa). (**b**) Work per cycle relating to the stability of the PU/PANI hybrid nanofibrous bundle (electrolyte = 1 M MSA, scan rate = 5 mV/s, potential vs. Ag/AgCl). Reprinted with permission from [109]. (**c**) Harmonic responses of the electrospun PANI/CA actuators powered by sinusoidal electrical inputs with an excitation frequency of 0.1 Hz and a peak voltage of 3 V; time history of tip displacements. (**d**) Hysteresis responses of current–voltage plots for PANI/CA actuators. Reprinted with permission from [27].

The electrochemical actuation profile of the PU/PANI nanofibrous bundles is presented in Figure 7a. The PU/PANI hybrid nanofibrous bundle responded to an electrical stimulus, resulting in an initial linear strain of 1.6% at an applied stress of 1.03 MPa. Those artificial muscle fiber bundles showed 2% of the actuation strain in over 100 cycles with the working efficiency of each cycle higher than 75% (Figure 7b). The PU/PANI nanofibrous bundle showed minor creep up to an 11 mN load (2.3 MPa), above which it showed significant creep behavior in actuation. Furthermore, by the incorporation of graphene, the actuation performance of this nanofiber slightly decreased, but its modulus significantly increased [109]. Hong et al. showed that PANI nanoparticles enhanced the actuation capacity of CA in an electrospun CA/PANI biocomposite film [27]. The harmonic responses for three electrospun actuators responding to sinusoidal electrical inputs with an excitation frequency of 0.1 Hz and a voltage amplitude of 3.0 V were measured (Figure 7c,d). The tip displacement of the 0.5 wt% PANI/CA biocomposite actuator was four times larger than in the case of the pure CA actuator, as shown in Figure 7c. Also, the increase in the addition of PANI from 0.1 wt% to 0.5 wt% improved tip displacement at all tested frequencies. This phenomenon can be explained by the combination of the orientation of permanent dipoles under applied electric current and polarization due to the displacement of ions and charge injection from electrodes [27].

An electroactive polymer actuator composed of poly(vinylidene fluoride) (PVDF) membranes enhanced with bacterial cellulose nanowhiskers (BCNWs) was developed using electrospinning [110]. Its actuating performance was reported as ±3.4 mm and 4.5 mm for the sinusoidal and step inputs. 

**Figure 8:**
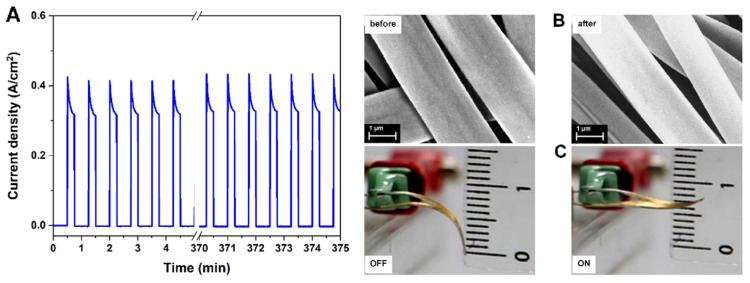
Artificial muscle lifetime. (**A**) The current density registered for 500 actuation cycles. (**B**) SEM images of the metalized electrospun fiber network attached on a PDMS sheet before and after 500 ON/OFF voltage cycles. (**C**) Snapshots taken after 500 actuation cycles of the artificial muscle. Reprinted with permission from [113].

PPy has been frequently used to develop soft artificial muscles [111]. Beregoi et al. reported on PPy actuators based on Nylon 66 electrospun microribbons. The microribbons had one side coated with a gold layer and finally a conductive layer of PPy was added by means of electrochemical deposition [112]. The electroactive microribbons expanded and shrank by switching the applied current or potential. The conformational changes in the polymer chains was due to the insertion/expulsion of the ions from the electrolyte (NaCl) and electrons from the gold. Furthermore, the asymmetry and anisotropy of the PPy-covered microribbons led to irregular volume changes, inducing the straightening/twisting of the entire piece. Furthermore, these electrospun actuators were able to show a simultaneous actuation and sensitivity, which is essential for the complex activity of artificial muscles [13]. Bunea et al. presented a new architecture of an artificial muscle based on a gold-covered Nylon 66 microfiber electrode attached to a thin foil of PDMS. The PDMS-based artificial muscle showed excellent stability during 500 cycles of testing while applying a voltage of 2.2 V (Figure 8) [113].

Severt et al. synthesized aligned silk/PPy nanofibrous bundles using electrospinning and sequential chemical and electrochemical polymerization processes [36]. In the same study, poly(hydroxymethyl-3,4-ethylenedioxythiophene) (PEDOT-OH) was also electrochemically polymerized on the surface of silk nanofibers. Samples were doped with *para*-toluene sulfonic acid (*p*-TSA) or sodium dodecylbenzenesulfonate. The results revealed that the actuation stress and response time of the silk/PEDOT-OH nanofibrous actuator was better than that of the silk/PPy sample. Devices doped with *p*-TSA exchanged both anions and cations, with anion exchange being the dominant mechanism. 

**Figure 9:**
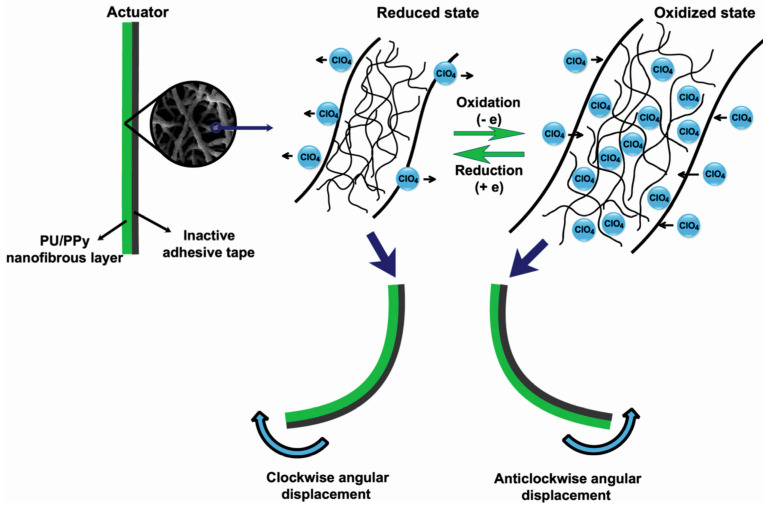
A schematic representation of the bending actuation mechanism of the PU/PPy nanofibrous actuators during oxidation and reduction processes. Reprinted with permission from [26].

Recently, Harjo et al. coated PPy on the surface of glucose–gelatin nanofibers to prepare a linear bioactuator [114]. It should be underlined that PPy coated the glucose–gelatin nanofibers individually, not as a bulk phase. The nanofiber material exhibited electrochemomechanical activity in both aqueous and organic (PC) electrolyte solutions, with good conductivity (0.45 S·cm^−1^) as well as actuation strain (1.2%) and stress (3.15 kPa) values. Furthermore, 120 cycles of strain/stress changes revealed no significant loss of strain or stress. Another PU/PPy nanofiber actuator was prepared by Ebadi et al. During cyclic voltammetry responses, PU/PPy actuators consumed total charges of 2.0–5.0 C in a 0.1 M LiClO_4_ electrolyte solution with potentials ranging between −0.6 and 0.8 V [26]. The mechanism of the actuation process explained by the authors is shown schematically in Figure 9. During the oxidation process, the electrons were extracted from the PPy, whereas in the reduction process, electrons were injected into it. Therefore, the perchlorate anions and water molecules entered the polymer structure from the electrolyte solution during the oxidation process of the actuators and left the polymer structure during the reduction process to balance the electric charge and osmotic pressure. This phenomenon resulted in a change in the volume, and therefore, in the bending angle of the produced actuator due to the mechanical constraints imposed by the inactive adhesive tape (Figure 9).

**Figure 10:**
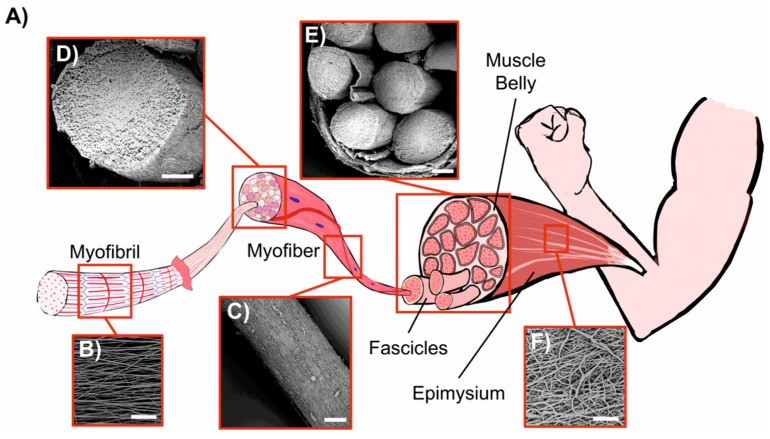
Comparison between biological skeletal muscle and electrospun PU structures. (**A**) Schematic of the hierarchical structure of skeletal muscle. (**B**) Mat of aligned nanofibers. eA single nanofiber corresponds to a myofibril (scale bar = 20 µm). (**C**) Bundle of aligned fibers, corresponding to a myofiber (scale bar = 100 µm). (**D**) Cross-section of an aligned bundle, showing the parallel arrangement of the inner nanofibers (scale bar = 100 µm). (**E**) Cross-section of the hierarchical nanofibrous electrospun structure (HNES), compared to the cross-section of a biological muscle belly (scale bar = 150 µm). (**F**) HNES membrane, resembling the epimysium membrane that envelops the muscle belly (scale bar = 100 µm). Reprinted with permission from [115].

Gotti et al. prepared PU/PPy-based hierarchically arranged nanofibrous structures similar in architecture and passive mechanical properties to skeletal muscles. The structure of the obtained electrospun membrane was packed in a form of bundles taut together as the biologic membrane epimysium in skeletal muscle (Figure 10) [115].

## 6. Electrospun Actuators Based on Liquid Crystalline Elastomers (LCEs)

**Figure 11:**
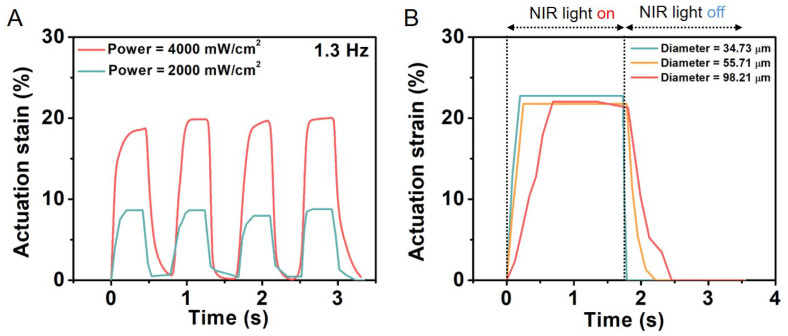
The response of PDA-coated LCE microfibers with different diameters exposed to near-IR light of two different powers. (**A**) Actuation strain of PDA-coated LCE fiber as function of time under two laser beams with different powers. (**B**) The response speed of PDA-coated LCE fiber with different diameters. Reprinted with permission from [116].

LCEs constitute a class of materials that possess both elastic properties as conventional elastomers and anisotropic physical properties due to their liquid crystalline state of order. LCE-based microfiber actuators based on the nematic–isotropic phase transition of liquid crystal mesogens were reported to generate a large actuation strain of 60% with a simultaneous fast response <0.2 s and a high power density of 400 W/kg. Furthermore, when coated with a polydopamine layer, the actuation of the electrospun LCE microfiber could be precisely and remotely controlled by a near-IR laser through the photothermal effect. As a result, He et al. were able to successfully construct a microtweezer, a microrobot, and a light-powered microfluidic pump using the electrospun LCE microfiber actuator (Figure 11) [116]. Krause et al. obtained electrospun crosslinked nematic fibers with a uniform alignment [19]. These highly oriented fibers exhibited a liquid crystalline state under ambient conditions.

**Table 3:** Overview of electrospun electroactive and LCE-based actuators.

Composition and Design	Morphology	Fabrication of Nonwovens	Electrical and Mechanical Properties	Biocompatibility	Applications	Ref.
CA matrix with PANI nanoparticles (0.1 wt% or 0.5 wt%) dispersed	Membranes’ thickness: 100 µm	Electrospinning: Needle inner diameter: 0.21 mm; Applied voltage: 20 kV; Distance to collector: 20 cm; Solution flow rate: 2.0 mL/h; Solution concentration: 20 wt/v% in DMAc/acetone 2:1 *v*/*v*	Ionic conductivity: 8.7·10^−4^ S·cm^−1^ in pure CA, 10.6·10^−4^ S·cm^−1^, and 19.2·10^−4^ S·cm^−1^ in 0.1 wt% and 0.5 wt% CA/PANI samples, respectively	Fibroblast cell line (NIH/3T3) attachments and spreading over the electrospun membranes were observed	A dry PANI/CA bio-composite actuator showing electrically driven bending deformations	[27]
CA matrix with fullerenol (0.1 wt% or 0.5 wt%) dispersed	Nanofibers’ diameter range: 400–800 nm; Membranes’ thickness: 100 µm	Electrospinning: Solution (20 wt/v%) of cellulose acetate in DMAc/acetone (2:1 *v*/*v*) with 0.1 or 0.5 wt% fullerenols; Needle inner diameter: 0.21 mm; Applied voltage: 25 kV; Distance to collector: 15 cm; Solution flow rate: 2.0 mL/h	Ionic conductivity: 8.2·10^−4^ S·cm^−1^ in CA, 11.5·10^−4^ S·cm^−1^, and 18.1·10^−4^ S·cm^−1^ in 0.1 wt% and 0.5 wt% CA/fullerenol samples; Tensile strength of CA fibers: 1.6 MPa; Tensile strength of CA fibers with 0.5 wt% fullerenol: 2.75 MPa	All electrospun nanofibers did not show any inhibition of *Escherichia coli* K-12 bacteria on agar plates, indicating a good biocompatibility of the membranes	Biocompatible actuators	[97]
PVA/PANI nanofibers deposited on interdigitated electrodes	Fiber diameter between 100 and 200 nm; density of nanofibers: 10^6^ nanofibers per square centimeter	Electrospinning: Applied voltage: 20 kV; Distance to collector: 13 cm; Solution flow rate: 0.5 mL/h; Solution concentration: 4% or 5% (*v*/*v*) in water; Speed of collector: 400 rpm	Electrical conductivity: 4% solution: 10^−5^ S·m^−1^; 5% solution: 3·10^−6^ S·m^−1^	No data	Gas sensor for ammonia detection	[90]
Composite meshes of PANI and well-blended PLCL/SF with NGF incorporated	Fiber diameter between 683 ± 138 and 411 ± 98 nm; average thicknesses of meshes were between 0.032 and 0.037 mm	Electrospinning of PLCL/SF/PANI nanofibers: Needle inner diameter: 0.51 mm; Applied voltage: 12 kV; Distance to collector: 5–6 cm; Solution flow rate: 1.0 mL/h; Solution concentration: 4% (*v*/*v*) in water; Speed of collector: 4000 rpm NGF-loaded PLCL/SF/PANI core–shell fibers were fabricated by coaxial electrospinning	Electrical conductivity: 30.5 ± 3.1 mS cm^−1^; Tensile strength from 5.7 ± 0.9 MPa to 13.7 ± 1.5 MPa depends on composition	Effective support of rat pheochromocytoma 12 (PC12) neurite outgrowth, increased percentage of neurite-bearing cells and the median neurite length; enhanced proliferation and decrease in the toxicity effect of PANI in Schwann cells	Electrical stimulation and nerve growth factor (NGF) on neuron growth	[91]
Blends of PANI and PLCL	Fiber diameter between 100 and 800 nm	Electrospinning of PANI/PLCL: Solution concentration (*v*/*v* %): 15:85 in HFP (0.515 g/mL); Needle inner diameter: 0.337 mm; Applied voltage: 18–20 kV; Distance to collector: 20 cm; Solution flow rate: 20 µL/min; Speed of collector: 2600 rpm	Electrical conductivity: about 0.00641 S·cm^−1^	Based on viability tests, morphological changes, and expression of differentiation proteins in PC12 cells, PANI/PLCL fibers enhanced the NGF-induced neurite outgrowth of PC12 cells	Development of electrically conductive, engineered nerve grafts	[92]
PEDOT nanofibers obtained on the electrospun PVP	Fiber diameter of 350 ± 60 nm	(1) Electrospinning of PVP: Needle inner diameter: 0.58 mm; Applied voltage: 27 ± 1 kV; Distance to collector: 15 cm; Solution flow rate: 1.0 mL/h; Solution concentration: 1.0 and 1.5 wt% in iron(III) p-TS 40 wt% in butanol (2) In situ vapor-phase polymerization of EDOT (3) Removal of PVP	Electrical conductivity: 60 ± 10 S·cm^−1^	No data	Electronic devices requiring flexibility and/or significant surface area, such as sensors or energy storage systems	[94]
The PVA/PANI hybrid mat consisted of PANI nanostructures grown on the surface of individual nanofibers; in the wet state, mats were rolled up conveniently into a multilayered cylindrical structure	Diameter of PVA nanofibers: 450 nm; individual PVA/PANI fibers: 1.2 µm diameter of PANI nanostructures: <70 nm	(1) Electrospinning of PVA: Applied voltage: 10 kV; Distance to collector: 12 cm; Solution flow rate: 10 μL/min; Solution concentration: 7.5 wt% PVA in water (2) In situ chemical polymerization of aniline on PVA mats	Electrical conductivity: 2.35 S·cm^−1^	No data	For fabricating high-performance electrochemical actuators	[95]
PANI/gelatin fibers	Fiber diameter decreased from 803 ± 121 nm for pure gelatin fibers to 61 ± 13 nm for 60:40 PANI–gelatin blend fibers.	Electrospinning of PANI/Gelatin: Volume ratios of PANI:gelatin were 0:100, 15:85, 30:70, 45:55, and 60:40. The following concentrations (*w*/*v*) of the solutions in HFP were 8.00, 6.85, 5.69, 4.54, and 3.38%, respectively. Applied voltage: 10 kV; Distance to collector: 10 cm	Sample with 45:55 ratio of PANI to gelatin; Tensile strength: 10.49 ± 0.96 MPa; Elongation at break: 0.09 ± 0.03%; Tensile modulus: 1384 ± 105 MPa; Conductivity (S·cm^−1^): 0.005, 0.01, 0.015, 0.017, and 0.021 for volume ratios of PANI to gelatin of 0:100, 15:85, 30:70, 45:55, and 60:40, respectively	PANI–gelatin blend fibers supported H9c2 rat cardiac myoblast cell attachment and proliferation to a similar degree as the control tissue culture-treated plastic (TCP) and smooth glass substrates	“Intelligent” biomaterials for cardiac and neuronal tissue engineering	[98]
SF scaffolds coated with PPy	PPy-SF mesh: 80–90 µm thickness	(1) Electrospinning of SF: Needle inner diameter: 0.45 mm; Applied voltage: +6 kV was applied to the capillary tube and −5 kV to the collector; Distance to collector: 10 cm; Solution flow rate: 6.0 mL/h; Solution concentration: 17 wt/v% in HFIP; (2) Polymerization of pyrrole on the SF meshes	Young’s modulus range: 266.7 ± 17.3 MPa for the SF meshes and 310.5 ± 37.6 MPa for the PPy-SF meshes; Voltametric responses ranging between 10 and 0.5 mV·s^–1^	Uncoated and PPy-coated materials support the adherence and proliferation of adult human mesenchymal stem cells (*ah*MSCs) or human fibroblasts (*h*Fbs)	Biocompatible actuators	[100]
Nanofibrous PU/PPy	Thickness of the PU, PU/PPy-ClO_4_, PU/PPy-pTS, and PU/PPy-TFSI nanofibers: 10 ± 1, 24 ± 2, 23 ± 2, and 43 ± 3 µm, respectively; Diameter of PU/PPy nanofibers: 719 ± 74 nm, 571 ± 73 nm, and 556 ± 77 nm, respectively, for ClO_4_, pTS, and TFSI dopants.	(1) Electrospinning of PU: Needle inner diameter: 0.718 mm; Applied voltage: 14 kV; Distance to collector: 25 cm; Solution flow rate: 0.3 mL/h; Speed of the collector: 5 rps; Solution concentration: 7 wt/v% in DMF (2) Polymerization of pyrrole on the PU meshes	The electrical conductivity of PU/PPy nanofibers produced using ClO_4_, pTS, and TFSI dopants was measured to be 158, 277, and 315 S·cm^−1^. In LiTFSI electrolyte solution, the PU/PPy nanofibrous artificial muscle achieved a bending displacement of 720° in a potential cycle between −0.8 and +0.8 V.	No data	Nanofibrous artificial muscles	[102]
PU/PPy-pTS nanofibers	Diameter of PU nanofibers: 221 ± 30 nm; Coated nanofibers: 566 ± 67 nm	(1) Electrospinning of PU: Applied voltage: 10, 12, and 14 kV; Distance to collector: 15, 20, and 25 cm; Solution flow rate: 0.3 mL/h; Solution concentration: 7, 8, and 9 wt/v% in DMF (2) Polymerization of pyrrole and sodium p-TS on the PU meshes	Conductivity of 276.34 S·cm^−1^; Reversible angular displacement capability about of 141°	No data	Artificial muscles	[103]
PANI/Au microtubes	The inner diameters of PANI/Au microtubes in the range of 1.2–1.5 µm	(1) Electrospinning of PMMA: Applied voltage: 20 kV; Distance to collector: 17 cm; Solution flow rate: 0.5 mL/h; Solution concentration: 10 wt/v% in DMF; Collector rotation speed: 2000 rpm (2) Coating fibers with Au (3) Electrochemical PANI deposition process (4) Removal of PMMA by immersing in DCM	By switching the voltage between −0.2 and 1 V, PANI-coated microtubes could reversibly bend	No data	Artificial muscles	[108]
PU/PANI hybrid nanofibrous bundle	Diameter of individual hybrid nanofibers in the bundles: about 900 nm; Average diameter of PU/PANI hybrid nanofibrous bundle: about 90 µm; Average diameter of PU nanofiber: about 400 nm; Thickness of PANI coating: about 250 nm	(1) Electrospinning of PU: Needle inner diameter: 0.337 mm; Applied voltage: +7 kV in the capillary tube and −5 kV in the collector; Distance to collector: 13 cm; Solution flow rate: 4.0 µL/min; Solution concentration: 10 wt% in chloroform (2) In situ chemical polymerization of aniline	Conductivity of 0.5 S·cm^−1^	No data	Nanofibrous artificial muscles	[109]
PVDF with 0.05 wt% and 0.1 wt% BCNW	Thickness of the PVDF membrane: 215 µm; PVDF-BCNW composites: 176 µm and 151 µm, respectively, for 0.05 and 0.1%	Electrospinning of PVDF: Needle inner diameter 0.838 mm; Applied voltage: 12 kV; Distance to collector: 24 cm; Solution flow rate: 1.5 mL/h; Solution concentration: 25 wt% in DMF and acetone (1:1, *v*/*v*)	PVDF/BCNW (0.1 wt%) actuator had a fast response and large tip displacement. Young’s modulus and yield strength around 3.5 GPa and around 9.5 MPa, respectively.	No data	Actuators anticipated in the fields of biomimetic robotics, medical devices, various actuators, and sensors	[110]
Free-standing Nylon-6/6 PPy-coated microribbons	Widths of the gold-coated electrospun microribbons: 1–1.5 µm; Thickness of the PPy layer: ~80 nm	(1) Electrospinning of Nylon-6/6: Needle inner diameter 0.838 mm; Applied voltage: 25 kV; Distance to collector: 15, 20 cm; Solution flow rate: 0.05–0.10 mL/h; Solution concentration: 22 wt% in formic acid (2) Coating of fibers with Au (3) Electrochemical PPy deposition process	The fabricated actuator responded by curling and straightening when the external stimulus current, pH, and temperature was applied	No data	Soft actuators sensing different external stimuli, bifunctional electrochemical devices	[111,112]
Au/Nylon-PDMS - Au covered Nylon-6/6 micrometric fibers attached to a thin PDMS film	Average diameter of the fibers: 2.08 ± 0.1 µm; Total thickness of the device: 250 ± 2.5 µm	(1) Electrospinning of Nylon-6/6: Needle inner diameter 0.838 mm; Applied voltage: 20 kV ± 2 kV; Distance to collector: 15 cm; Solution flow rate: 0.2 mL/h; Solution concentration: 30 wt% in formic acid (2) Coating fibers with Au (3) Assembly of metalized fiber network to the PDMS sheet	Displacement of 0.8 cm when applying 2.2 V (500-cycle test performed)	No data	Artificial muscle	[113]
Glucose–gelatin nanofiber scaffolds chemically coated with PPy	PPy covered individual fibers separately, resulting in uniformly coated fibers with a similar diameter of 1.58 ± 0.1 µm in aqueous electrolyte and 1.43 ± 0.12 µm in PC electrolyte	(1) Electrospinning of glucose/gelatin (in 1:10 wt% ratio, dissolved in 10 M acetic acid): Applied voltage: 17.5 kV; Distance to collector: 14.5 cm; Solution flow rate: 5–7 µL/min; (2) Crosslinking the nonwovens at 175 °C (3) Electrochemical PPy deposition process	PPy coated the CFS fibers showing electrochemomechanical activity in both aqueous and organic (PC) electrolyte solutions; In water: conductivity: 0.45 ± 0.034 S·cm^−1^; reversible strain and stress of 1.2% and 3.15 kPa, respectively.	No data	Wearable devices, such as e-skin or in soft robotics devices	[114]
Poly(ether-ester-urethane) (PU): poly [4,4′-methylenebis(phenyl isocyanate)-*alt*-1,4-butanediol/di(propylene glycol)/polycaprolactone]	Mean diameter: 0.88 ± 0.36 µm; Volume fraction: 0.47 ± 0.08; Bundles were homogeneous (diameters of random bundles 468 ± 33 µm and aligned ones 419 ± 37 µm without the presence of beads)	Electrospinning of PU: Four needles with inner diameter of 0.51 mm; Applied voltage: 23 kV; Distance to collector: 18 cm; Solution flow rate: 0.3 mL/h; Solution concentration: 25 *w*/*v* in THF:DMF (70:30, *v*/*v*) collector, with a speed of 1500 mm min^–1^	A failure force of the random mats: F_F_ = 0.83 ± 0.08 N (εF = 232 ± 17%); Of bundles: F_F_ = 0.50 ± 0.08 N (εF = 182 ± 18%)	No data	Muscle tissue engineering and soft actuators	[115]
RM 257 as a liquid crystal mesogen and HDT as a chain extender doped with PDA	The diameters of microfibers ranged from 10 to 100 µm	(1) Electrospinning of ink (RM257, HDT, HHMP): Four needles with inner diameters of 1.194 mm; Applied voltage: 6 kV; Solution flow rate: 0.02 mL/min; Solution concentration: 20 wt% in TCM During the electrospinning process, the LCE microfibers were exposed to UV light (365 nm wavelength) to trigger the crosslinking reaction (2) Preparation of PDA-coated LCE microfibers through a simple dip-coating process	Longitudinal contraction under the exposure of NIR light of PDA-LCE; Actuation strain: >50%; actuation stress: 0.3 MPa; response speed: 300%/s; and work density: 20 kJ/m^3^ The temperature actuation strains were 55, 33, and 30% at 120 °C when the applied stresses were 0.02, 0.08, and 0.16 MPa, respectively	No data	LCE microfiber actuator for artificial muscles, microrobots, or microfluidic pumps.	[116]

Abbreviations: CA: cellulose acetate; PANI: polyaniline; PVA: poly(vinyl alcohol); NGF: nerve growth factor; PLCL: poly[(L-lactide)-co-(ε-caprolactone)]; SF: silk fibroin; PEDOT: poly(3,4-ethylenodioxythiophene); PVP: polyvinylpyrrolidone; EDOT: 3,4-ethylenedioxythiophene; PPy: polypyrrole; p-TS: p-toluenesulfonate; PU: polyurethane; PMMA: poly(methyl methacrylate); BCNW: bacterial cellulose nanowhiskers; PDMS: polydimethylsiloxane, HDT: hexane dithiol; HHMP: (2-hydroxyethoxy)-2-methylpropiophenone; DPA: polydopami.

## 7. Summary and Outlook

Electrospinning has emerged as a modern and versatile fabrication technique, offering unique advantages for the production of advanced materials with diverse applications. This review highlights the fascinating field of bioinspired electrospun polymer actuators, focusing on three major categories: hydrogels, shape-memory polymers (SMPs), and electroactive polymers [1]. These advanced materials hold tremendous promise in various applications, ranging from soft robotics and biomedical devices to energy harvesting and artificial muscles [7].

Through the ingenious combination of electrospinning and hydrogel technology, intricate fibrous structures are crafted, resulting in actuators that exhibit remarkable shape-changing abilities in response to external stimuli. The actuation in electrospun hydrogel actuators is inspired by the skin of human hands [51] and pine cones [52], which provide reversible movements based on swelling/deswelling effects in a bilayered structure. The active layer of the actuators can uptake water, resulting in volume increase and the passive layer is inert to dimensional change. These bilayer structures based on electrospun hydrogel meshes have enabled bending movements, which rely on internal mechanical stresses generated at the interface of the two layers. Furthermore, along with sensitivity to water, swelling/deswelling of electrospun hydrogel systems triggered by other stimuli such as heat, light, electric field, or pH sensitivity have also been introduced to initiate the actuation mechanism. Here, inorganic filler such as iron oxide nanoparticles or functional polymers such as PNIPAM, polypyrrole, polyaniline, or polyacrylic acid have been used. Among different stimuli, heat is the most extensively studied stimulus and electrospun hydrogels based on PNIPAM are the most reported thermosensitive actuating systems. However, the activation temperature of these actuators is restricted to the LCST of PNIPAM. Here, a future direction could be the electrospinning of other thermosensitive polymers with a range of LCST, such as hydroxypropyl cellulose, poloxamers, or poly(vinyl phosphonate), etc. Nevertheless, in order to realize complex shape shifts, the creation of different fiber orientations is recommended or subsequential 3D printing on electrospun membranes will have to be performed. In addition, when another kind of movement is mandatory, new material design is required for these bilayered structures. One concept to create hydrogel actuators providing complex movements is the integration of shape-memory functionality in hydrogel systems. Here, the challenge would be the combination of stimulus-sensitive domains with swelling domains, in which the two different types would act independently of one another (swelling would not influence the stimulus-sensitive domains and stimuli would not influence the swelling domains). One approach to create electrospun shape-memory hydrogel actuators could be the co-continuous electrospinning of semi-crystalline SMPs and hydrogels followed by post curing to enable interpenetrating polymer networks. In this case, SMPs would enable the actuation behaviour and hydrogels would act as the swelling segment. In contrast to the actuation mechanism of bilayered structures, here, the crystallization/melting of crystallizable domains of SMPs would enable reversible movements. Nevertheless, despite the promising capabilities of electrospun actuators based on hydrogels, several challenges loom ahead. For instance, selecting hydrogel materials that can be effectively electrospun, while retaining their responsive properties, is a complex task, as not all hydrogels can withstand the electrospinning process. Hydrogels are known for their soft nature, so creating electrospun actuators that exhibit both responsive behavior and mechanical robustness is intricate [62].

SMP fibrous meshes have shown immense potential and exciting prospects for the cutting-edge technology in the field of soft robotics and beyond [72]. However, only a few studies related to electrospun SMPs enabling a reversible actuation have been reported. Electrospun actuators based on a crosslinked PCL resulted in an actuation performance of *ε*’_rev_ = 15%, while for PEVA-based actuators, their value of *ε*’_rev_ could vary in a range from 10 ± 1% to 17 ± 2% depending on the alignment of the fibers [76]. The post curing of PCL and PEVA fibers by exposure to UV light could be avoided by blending a multiblock copolymer based on PLLA and PCL (PLLA-PCL) with oligo(D-lactide) (ODLA). Here, the stereocomplex formation between the opposite enantiomers of PLA enabled physical crosslinks in the blend [77]. The fibrous meshes of the PLLA-PCL/ODLA blend resulted an actuation magnitude of *ε*rev = 7.8 ± 0.8%, which could be increased to 15 ± 1.8% by twisting the electrospun fibers into a yarn [78]. Here, achieving reproducibility and scalability remains a challenge. Furthermore, in contrast to hydrogel-based actuators, SMP fibrous meshes require a thermomechanical programing procedure to align their crystalline domains in a certain direction and subsequent heating and cooling cycles to enable the actuation process. To avoid this thermomechanical programming procedure, new studies related to the in situ alignment of crystalline domains during the electrospinning process resulting in oriented fibrous meshes would be required. SMP-based fibrous meshes have generally poor mechanical properties. The addition of inorganic nanofillers could be helpful to improving their mechanical properties. Nevertheless, ensuring robust mechanical properties of electrospun SMPs while maintaining their actuation behavior is a delicate balance. The electrospinning process can be intricate and sensitive to parameters, leading to inconsistencies in fiber morphology, size, and distribution. The characterization of electrospun SMPs, particularly at the nano scale, demands sophisticated and precise techniques. Developing non-destructive, high-resolution methods to study structure–property relationships is crucial. Addressing these challenges will require concerted efforts from researchers, engineers, and industry stakeholders to unlock the full potential of electrospun SMPs and transform them into practical and impactful materials for a wide array of applications.

Electrospun actuators harnessed from conductive polymers (CPs) leverage the unique electroactive properties of polymers, responding to electrical stimuli with dynamic shape changes. However, due to poor physical features induced by insolubility, infusibility, and brittleness, the applicability of CPs as actuators requires significant challenges to be addressed. As the utilization of electrospinning requires organic solvents for the preparation of solution, therefore, the synthesis of new CPs by the modification of existing CPs or synthesis of new derivatives with improved solubility and spinnability is mandatory. Furthermore, it is important to further improve the conductivity and thermal stability of electrospun CP fibers. This can be performed by the fabrication of CP-based electrospun composites containing a variety of nanofillers, such as inorganic metal oxides, CNTs, graphene, carbon nanotubes, metal or semiconductor nanoparticles, and quantum dots. It has been shown that preparation of polymer-based composites such as Au-doped poly PAN–PANI core–shell nanofibers [117], silver-doped PVDF fibers [118], or PVA/polyurethane/Au [119] can improve these properties and lead to better applicability due to better conductivity or thermal stability [120].

Moreover, finding or synthesizing better organic solvents or functional doping agents would be a promising direction, which is critical for realistic applications. Nevertheless, there is severe concern about the application of electrospun CPs in the medical field, as, for example, biosensors, bio-actuators, and tissue engineering scaffolds, due to the risk of cytotoxicity caused by residual solvent and solvent accumulation. Due to this reason, the use of melt electrospinning to produce fibers with the reproducible diameter and structure of electrospun 3D materials, seems to be a promising perspective [81,121,122]. However, CPs are difficult to melt into electrospun fibers, which opens new ways for post treatments or combined methods of fiber preparation.

Considering the present state of the art, a great challenge is to reach a large-scale production of electrospun fibers and 3D materials and their commercialized applications. The electrospinning process is still mostly investigated in academic laboratories with the usage of single-needle electrospinning devices. Therefore, more attention should be paid to the development of this technique, which would enable various multi-needle/multi-jet electrospinning and nozzle-less electrospinning techniques [123,124]. However, dense arrays of needles in multi-nozzle designs implemented to increase the scalability of electrospinning create repulsion and block nozzles, impacting their scalability. Therefore, more efforts in finding alternative techniques should be made. Furthermore, the potential of hybrid materials that combine stimulus-sensitive hydrogels, shape-memory polymers, and electroactive polymers is particularly emphasized, as such combinations may unlock unprecedented actuation capabilities and multifunctionality. Moreover, the integration of emerging technologies like nanotechnology and additive manufacturing could further enhance the performance and efficiency of these actuators. In the biomedical field, the development of biocompatible and bioresorbable materials opens up exciting possibilities for implantable devices and tissue engineering applications. Collaboration between different disciplines, such as materials science, engineering, and biology, will be crucial in overcoming challenges and accelerating the translation of these bioinspired actuators from the laboratory to real-world applications. In conclusion, this review paper serves as a comprehensive resource for researchers and enthusiasts interested in the cutting-edge developments of bioinspired electrospun polymer actuators. By fostering collaboration and innovation, this field holds the potential to revolutionize numerous industries and bring us closer to achieving highly adaptable, bioinspired systems with a wide array of practical applications.

## Figures and Tables

**Table 2:** Overview of electrospun shape-memory polymer (SMP) actuators.

Stimulus	Materials and Solvents	Morphology	Electrospinning Conditions	Actuation Capability (ε’_rev_)	Applications	Ref.
Heat	PCL crosslinked using UVcrosslinker (TAIC and BP); Solvents: chloroform and ethanol (7:3 volume ratio)	Fiber dimeter: before crosslinking (2.3 ± 0.6 µm) and after crosslinking (2.2 ± 0.7 µm)	Voltage: 20 kV; distance: 30 cm; rotational speed of collector: 1 rpm	6 ± 1% at ε_m_ = 100%; 22 ± 1% at ε_m_ = 300%	Smart membrane materials for textiles and filtration	[76]
Heat	PEVA crosslinked using UV crosslinker (TAIC and BP); Solvent: chloroform	Fiber diameter: 15.2 ± 2 µm for random and 9 ± 2 µm for aligned fibers; Mesh thickness: ~100 µm	Voltage: 8 kV; feed rate: 2.4 mL/h; distance: 10 cm; rotational speed of collector: variable 1–180 rpm	17 ± 2% (random); 12 ± 2% (stacked); 10 ± 1% (aligned)	Soft robots, tissue regeneration	[77]
Heat	Multi-block copolymer containing PLLA and PCL segments (PLLA-PCL) blended with ODLA; Solvent: tetrahydrofuran (THF)	Fiber diameter: 1.8 ± 0.6 µm; Mesh thickness: 50 ± 2 µm	Voltage: 10–13 kV; feed rate: 2.1 mL/h; distance: 10 cm; rotational speed of collector: 10 rpm	7.8 ± 0.8% for electrospun mesh; 15 ± 0.8% for twisted yarn	Soft robotics, medicines	[78]

Abbreviations: PCL (poly(ε-caprolactone); PEVA (poly(ethylene-co-vinyl acetate); TAIC (triallyl isocyanurate); BP (benzophenone); PLLA (poly(L-lactide)); ODLA (oligo(D-lactide)).
